# Recent Advances and Application of Machine Learning for Protein–Protein Interaction Prediction in Rice: Challenges and Future Perspectives

**DOI:** 10.3390/proteomes13040054

**Published:** 2025-10-27

**Authors:** Sarah Bernard Merumba, Habiba Omar Ahmed, Dong Fu, Pingfang Yang

**Affiliations:** State Key Laboratory of Biocatalysis and Enzyme Engineering, School of Life Sciences, Hubei University, Wuhan 430062, China; sarah@stu.hubu.edu.cn (S.B.M.); habbyommy128@gmail.com (H.O.A.)

**Keywords:** protein–protein interaction, machine learning, rice, deep learning, multi-omics integration, proteoforms

## Abstract

Protein–protein interactions (PPIs) are significant in understanding the complex molecular processes of plant growth, disease resistance, and stress responses. Machine learning (ML) has recently emerged as a powerful tool that can predict and analyze PPIs, offering complementary insights into traditional experimental approaches. It also accounts for proteoforms, distinct molecular variants of proteins arising from alternative splicing, or genetic variations and modifications, which can significantly influence PPI dynamics and specificity in rice. This review presents a comprehensive summary of ML-based methods for PPI predictions in rice (*Oryza sativa*) based on recent developments in algorithmic innovation, feature extraction processes, and computational resources. We present applications of these models in the discovery of candidate genes, unknown protein annotations, identification of plant–pathogen interactions, and precision breeding. Case studies demonstrate the utility of ML-based methods in improving rice resistance to abiotic and biotic stresses. Additionally, this review highlights key challenges like data limits, model generalizability, and future directions like multi-omics, deep learning and artificial intelligence (AI). This review provides a roadmap for researchers aiming to use ML to generate predictive and mechanistic insights on rice PPI networks, hence helping to achieve enhanced crop improvement programs.

## 1. Introduction

Protein–protein interactions (PPIs) are at the center of understanding molecular function and regulation within cells [1]. These interactions control various biological processes, such as signal transduction, gene expression regulation, metabolic pathways [2], and responding to stresses [3]. Insights into PPIs could facilitate not only the explanation of the molecular basis of such processes but also the identification of possible drug targets and the improvement in crop qualities [4,5]. The rice genome includes around 40,000–50,000 genes as reported by Krishna et al. [6], of which each has the potential to produce a variety of proteins. These proteins can interact with one another in complex networks to regulate rice growth, development, and response to environmental stimuli [7,8]. Rice interactome, made up of PPIs, is important in key functions like nutrients uptake, defense against pathogens, and tolerance to stresses [9,10].

Similar to other organisms, rice proteins exist in multiple proteoforms due to alternative splicing, sequence variation, and post-translational modifications (PTMs). These proteoforms influence how rice proteins behave in stress responses, development, and signaling. Different proteoforms can interact with distinct protein partners rewiring cellular signaling pathways, thereby adding layers of complexity to PPIs by altering interaction affinities and specificities in rice-specific contexts [11,12,13]. For example, in rice, proteoforms arising from PTMs have been shown to modulate responses to cold stress by altering protein stability and interactions, as seen in the regulation of OsHAG702-mediated cold tolerance [14]. Understanding these proteoform-dependent interaction networks not only deepens our knowledge of rice biology but also offers practical avenues for breeding and engineering rice varieties with improved resilience and stress tolerance.

Likewise, proteolytic proteoforms act as elusive components in hormonal pathways, influencing signaling cascades that affect PPI dynamics [15]. In plants broadly, proteoforms under environmental stress enable adaptive mechanisms, such as enhanced resistance to abiotic factors, by fine-tuning protein functions without requiring new gene expression [16]. This proteoform-level variability is particularly relevant to rice PPIs, where it contributes to the plant’s ability to rewire interactomes in response to biotic and abiotic challenges, as highlighted in studies on alternative proteoforms in plant assemblies [17]. Incorporating proteoform considerations into machine learning (ML) and deep learning (DL), are used to predict proteoform properties, identify novel proteoforms, and interpret their biological functions from raw experimental data.

Conventional techniques used to detect PPIs, including Yeast two-hybrid screening, Co-immunoprecipitation, and Pull-down assays, while successful, have a number of shortcomings [18,19,20]. They are time-consuming, labor-intensive, and tend to be very resource-demanding. Meanwhile, the scalability of experimental PPI identification is low in the context that it is not feasible to handle numerous proteins in parallel. In addition, PPIs are not fixed, and they may change over time due to various stimuli or environmental changes. To resolve these limitations in the era of high-throughput data, computational methods based on ML, have become increasingly important. These methods utilize different forms of biological data, such as protein sequences, 3D structures, genomic context, and functional annotations, to learn and predict PPIs with great precision. Therefore, ML-based methods such as Random Forest (RF) and Support Vector Machine (SVM), have been widely applied as a promising solution for predicting PPI at large scales [21,22,23,24,25,26].

Multiple strategies have been developed for predicting PPIs in rice. In a recent study, Zheng et al. [27] employed deep learning models to explore interactions between rice and pathogen proteins. Their approach successfully identified critical resistance genes like *PID2* and pathogen effectors such as AVR-Pik, offering valuable targets for breeding disease-resistant rice cultivars. One notable advancement is the use of structure-based approaches developed by Sun et al. [28], which introduced a docking-based method that leverages protein structural information to predict interactions, achieving high accuracy for proteins with known 3D structures. This approach has been particularly effective in mapping protein networks involved in rice development and other biologically complex pathways. Several studies have shown that ML-assisted PPI predictions could enable scientists to model rice proteome interactions, reveal concealed relationships among proteins, and prioritize genes for downstream analysis and breeding [28,29,30,31,32,33,34,35].

While several reviews have addressed computational and experimental approaches to PPIs in plants, few have specifically focused on the unique methodological advancements and biological insights offered by ML in rice. This review is focusing on rice, integrating traditional ML and emerging deep learning frameworks with rice-specific datasets like RicePPINet. It uniquely emphasizes interpretability, multi-omics integration, and practical applications in rice functional genomics and breeding, offering a critical synthesis of current capabilities, limitations, and future prospects for advancing rice systems biology and crop improvement. The objective is to provide a comprehensive and critical synthesis of ML-based PPI prediction efforts in rice, outlining the current capabilities, limitations, and future prospects of computational models for advancing rice systems biology and crop improvement.

## 2. Data Sources and Feature Engineering for PPI Prediction

### 2.1. Data Sources

The performance of ML models for PPI predictions is determined largely by the quality of training data. For rice, available resources are diverse but limited in coverage compared to model organisms. Key resources include general repositories like Search Tool for the Retrieval of Interacting Genes (STRING, version 12.0) and Biological General Repository for Interaction Datasets (BioGRID, version 4.4.420) provide crucial ground truth data but cover only a small fraction of the rice interactome [36,37]. To overcome the scarcity of experimental data, homology-based inference from Arabidopsis has been a common strategy for conserved pathways, with ~40% of interactions showing detectable conservation in rice [38]. A transformative advancement is the availability of rice-specific structural proteome data through AlphaFold2, enabling the large-scale extraction of structural features for interaction prediction [39]. Complementary omics data from resources like RiceFREND (version 2.0) and mass spectrometry further enrich training sets by adding functional context to structural predictions [40,41].

The scarcity of high-quality, experimentally validated PPIs necessitates rigorous strategies for dataset curation to build reliable benchmarks for model training and evaluation. A primary challenge is the selection of negative samples pairs of proteins that do not interact. Common approaches include random pairing from different subcellular compartments, which is simple but may include undiscovered true interactions, and the more biologically grounded method of selecting proteins with distinct localizations to make physical interaction unlikely [42,43]. Furthermore, the use of cross-validation schemes must be carefully considered. While k-fold cross-validation is standard, more robust methods like Leave-One-Protein-Out (LOPO) cross-validation provide a stricter test by holding out all pairs containing a specific protein, thereby assessing the model’s ability to predict interactions for novel proteins not seen during training [21,44].

Despite these strategies, the creation of a unified, high-confidence benchmark dataset for rice, integrating experimentally verified PPIs with carefully curated negative samples, remains a critical need for the community. Such a resource, used in conjunction with robust validation schemes, would significantly improve the comparability and biological relevance of ML-based PPI predictions. The unique strengths, data types, and coverage of these primary sources are systematically compared in Table 1.

### 2.2. Feature Selection

Effective feature selection is critical for accurate and interpretable PPI predictions (Table 2). Feature selection is the task of selecting the most relevant features and transforming raw data into meaningful representations for the model. Feature selection could ascertain the model to be more biologically interpretable and predictive by reducing noise and dimensionality.

#### 2.2.1. Sequence-Based Features

Sequence-based features form the foundation of input for most computational PPI prediction models when data on structure is normally not available. These features are obtained from amino acid sequences of proteins directly without invoking experimentally determined structural data, and are thus very accessible and computation-friendly. Amino acid composition (AAC) and derived descriptors such as CKSAAP and position-specific scoring matrices (PSSMs) remain widely used. While simple and scalable, they lack spatial context and often require complementary descriptors for improved accuracy [48,49].

#### 2.2.2. Structure-Based Features

Structure-based features capture spatial and physicochemical information that are essential to describe the nature of interaction interfaces. With more high-resolution three-dimensional (3D) protein structures available, structure-based features are more applicable in PPI prediction. The advancement of AlphaFold2, structural descriptors such as solvent accessibility, docking scores, and interface propensities have become feasible at proteome scale [28]. It is important to note that high-confidence AlphaFold2 models are now available for the vast majority of the rice proteome through dedicated databases, providing rice-specific structural data rather than relying solely on cross-species homology [50,51]. However, high computational cost and uncertainty in multi-protein complexes remain challenges.

#### 2.2.3. Function-Based Features

Function-based features offer a complementary perspective in PPI predictions by incorporating biological context into computational models. These features are often knowledge-driven and provide higher-level insights into the roles and relationships of proteins within cellular systems. Derived annotations from Gene Ontology (GO) terms, domains, and pathway membership add biological interpretability. Semantic similarity scoring of GO terms and domain–domain interaction data (e.g., Pfam, DOMINE) improve functional relevance but are limited by annotation completeness [52,53].

The integration of multiple feature types increasingly defines state-of-the-art approaches. Recent work suggests that embeddings from protein language models and graph-based encodings can unify sequence, structural, and functional information into richer feature spaces [54]. Such multi-modal representations will be essential for capturing the dynamic and condition-specific nature of rice PPIs.

**Table 2:** Common Feature Type Used in Rice PPI predictions.

Feature Type	Description	Advantages	Limitations	Typical Use in Rice PPI Modeling	References
Sequence-Based	Derived from primary amino acid sequence (e.g., AAC, CKSAAP, PSSM)	- Easy to compute - Requires no structural data - Useful for all proteins	- Limited to linear/local info - Misses spatial and contextual interactions	Used in SVM and RF models for rice phosphorylation and PPI predictions	[49,55,56,57]
Structure-Based	Based on 3D conformation: interface residues, solvent accessibility, dynamics	- Captures spatial interaction context - High biological relevance	- Requires high-quality 3D structures - Computationally expensive	Emerging in rice using AlphaFold2-based models; potential for DL integration	[28,54,58,59,60]
Function-Based	Biological annotations (GO terms, domains, co-expression, pathway membership)	- Provides functional and contextual insights - Improves biological interpretability	- Limited by annotation quality-May not generalize across tissues/stages	Used in GNN/DLNet models for network-based rice PPI inference	[7,53,61,62,63,64]

### 2.3. Evaluation Metrics

The evaluation of ML models used in PPI predictions is an important part of model construction and validation. Because complex and class-imbalanced biological data, particularly in PPI datasets with actual interactions overwhelmingly outnumbered by non-interacting pairs, careful selection and interpretation of evaluation metrics must be made for biological and computational relevance (Table 3).

Accuracy (Equation (1)) is a straightforward metric but can be highly deceptive in imbalanced PPI datasets. A naive model that predicts all pairs as “non-interacting” would achieve high accuracy yet fail to identify any true biological interactions, rendering it useless for discovery [65]. Therefore, metrics that focus on the correct identification of the positive class (interactions) are essential. Precision (Equation (2)) and Recall (Equation (3)) offer a more nuanced view. High precision is crucial when the cost of false positives is high, for instance, when prioritizing a shortlist of candidate interactions for costly experimental validation. It ensures that researchers are not wasting resources on false predictions [66]. High recall, on the other hand, is important for discovery-oriented tasks where the goal is to identify as many true interactions as possible from a pool, even at the risk of including some false positives [67]. The F1-score (Equation (4)) balances these two concerns as their harmonic mean and is a robust single metric for imbalanced datasets [68].

(1) $$Accuracy=\frac{TP+TN}{TP+TN+FP+FN}$$

(2) $$Precision=\frac{TP}{TP+FP}$$

(3) $$Recall=\frac{TP}{TP+FN}$$

(4) $$F1-Score=2\cdot \frac{Precision\cdot Recall}{Precision+Recall}$$

where TP = True positive, TN = True negative, FP = False positive and FN = False negative.

Another widely used measure is the Area under the Receiver Operating Characteristic curve (AUC-ROC) that evaluates a model’s discriminatory ability between interacting and non-interacting protein pairs at various classification thresholds [69]. However, in highly imbalanced scenarios, the ROC curve can present an overly optimistic view because the large number of true negatives (TNs) inflates the true negative rate (Specificity). For this reason, the Area Under the Precision–Recall Curve (PR-AUC) is often more informative and biologically meaningful for PPI prediction. The PR curve directly plots the trade-off between precision (y-axis) and recall (x-axis), completely ignoring the TN rate. This makes it particularly sensitive to the performance on the positive class. A high PR-AUC score indicates that the model can achieve both high recall and high precision, which is the ideal scenario for biological discovery: finding many true interactions with a high degree of confidence [42].

The Matthews Correlation Coefficient (MCC) (Equation (5)) is another robust metric for imbalanced data as it considers all four confusion matrix categories (TP, TN, FP, FN) and produces a high score only if the prediction is good across all of them [70]. An MCC value close to +1 indicates a near-perfect prediction, while 0 represents a random predictor. Its comprehensive nature makes it an excellent single-value metric for assessing the overall quality of a binary classifier in biological contexts.

(5) $$MCC=\frac{\left(TP\cdot TN\right)-(FP\cdot FN)}{\sqrt{\left(TP+FP)(TP+FN)(TN+FP)(TN+FN\right)}}$$

The class imbalance inherent in PPI prediction, where non-interacting pairs vastly outnumber true interactions, poses a significant challenge for ML models, often leading to biased classifiers that favor the majority class. To address this, recent methods employ both data-level and algorithm-level strategies. On the data level, sampling strategies such as Synthetic Minority Over-sampling Technique (SMOTE) generate synthetic positive instances to balance the dataset, while under-sampling can randomly remove negative instances, though at the risk of losing information [35,71]. At the algorithm level, class-weighted loss functions are a more sophisticated and widely adopted solution. These functions assign a higher cost to misclassifying a rare positive interaction during model training, thereby forcing the algorithm to pay more attention to the minority class.

For instance, models like DWPPI and DLNet implicitly handle imbalance through their architecture and training on large-scale networks, but explicitly incorporating a class-weighted cross-entropy loss can significantly boost the recall of true PPIs without sacrificing precision [72]. The use of ensemble methods like Random Forest, which aggregate multiple decision trees, also provides inherent robustness to imbalance. The choice of strategy is often validated by the subsequent improvement in robust metrics like MCC and PR-AUC, which, as discussed, are more informative than accuracy in such scenarios [67,73].

Beyond the choice of metrics, the validation strategy employed is equally important. K-fold cross-validation remains the most common approach, where the dataset is divided into k subsets and the model is iteratively trained and tested on different folds. This helps mitigate overfitting, particularly when data is limited. Stratified cross-validation, which maintains the class distribution across folds, is preferable in highly imbalanced datasets. Independent test sets, often drawn from entirely separate experimental batches or species, provide an additional layer of robustness and allow for the assessment of model generalizability. In rice PPI studies, cross-species validation using orthologous interactions from Arabidopsis has occasionally been employed, though such strategies can be biased if evolutionary conservation is also used as a feature.

Benchmarking models against baseline classifiers is another essential step. Simple methods such as random prediction, sequence similarity-based heuristics, or previously published models serve as reference points to evaluate improvements in predictive power. However, this process requires transparency in reporting preprocessing steps, negative sample generation, and statistical significance of performance gains. Negative samples in particular represent a major challenge in PPI prediction, as the absence of an interaction does not necessarily imply a true negative, only a lack of current experimental evidence.

Despite several publicly available resources, rice still lacks a universally accepted gold-standard PPI benchmark set. Datasets like RicePPINet, BioGRID, and STRING are often used, although with varying levels of curation and confidence. Tools such as PPIbench and PSICQUIC allow for large-scale benchmarking, but their integration with rice-specific data remains limited. Furthermore, the generation of negative samples and the potential presence of false negatives significantly complicate the evaluation process.

The absence of a standardized rice PPI benchmark dataset represents a significant hurdle for developing solid model evaluations. To mitigate this issue, we recommend developing a high-confidence rice PPI dataset through collaborations with such databases as RicePPINet and BioGRID by including experimentally verified interaction and curated negative samples. In addition, synthetic data generation or transfer learning from Arabidopsis could enhance the dataset coverage [47]. It would also be useful to have standardized protocols for preprocessing methods, negative sample selection, and metric reporting (including but not limited to MCC and PR-AUC with confidence intervals) to facilitate comparisons among studies. These standardization efforts would improve the confidence and biological relevance of ML-PPI predictions in rice crop.

In the future, the field would benefit greatly from the development of curated benchmark datasets and standardized evaluation protocols for rice PPI predictions. Emphasis should be placed on metrics such as MCC and PR-AUC that are better suited to imbalanced data. It should include confidence intervals or standard deviations across multiple runs to account for variability when reporting performances. Also, ML outcomes need to be complemented with biological validation or plausibility checks, such as evidence of co-expression, subcellular localization, or involvement in shared metabolic pathways. These will allow the predictive power of ML to be meaningfully aligned with biological relevance, ultimately contributing to more reliable functional genomics in rice.

**Table 3:** Common Evaluation Metrics Applied for Prediction of Rice PPIs.

Metric	Definition	Advantages	Limitations	References
Accuracy	Ratio of correctly predicted instances (TP + TN) to total predictions	Simple to compute and interpret; provides a general overview	Misleading in imbalanced datasets where negative class dominates	[65,73]
Precision	TP/(TP + FP)—proportion of positive predictions that are correct	Highlights model’s ability to avoid false positives	May ignore false negatives; not sufficient alone in imbalanced settings	[66,74]
Recall (Sensitivity)	TP/(TP + FN)—proportion of actual positives correctly identified	Important for identifying all true interactions; useful in biological discovery	Can be high even when precision is low; may lead to many false positives	[67,75]
F1-Score	Harmonic mean of precision and recall	Balances precision and recall; useful when class distribution is skewed	Does not consider true negatives; sensitive to threshold choice	[68,76]
AUC-ROC	Area under the receiver operating characteristic curve	Measures discrimination capability of model across all thresholds	Can be misleading in highly imbalanced datasets; less focused on the positive class	[69,77]
PR-AUC	Area under the precision–recall curve	Better reflects performance on imbalanced datasets; focuses on positive class	Sensitive to class imbalance and prevalence; interpretation may be less intuitive than ROC curves	[42,43]
Matthews Correlation Coefficient (MCC)	Correlation coefficient between observed and predicted binary classifications	Takes into account all elements of the confusion matrix; robust in imbalanced datasets	Less commonly used and harder to interpret; sensitive to dataset size	[70,78]

## 3. Machine Learning (ML) Methods for PPI Prediction

### 3.1. Traditional ML Methods

Traditional ML approaches, such as Support Vector Machines (SVMs), Random Forests (RFs), and k-Nearest Neighbors (kNN), have been developed and widely applied in PPI predictions at the early stage. They have advantages of interpretability, moderate computational cost requirement, and amenability in handling structured biological information. SVMs have been used most frequently in PPI predictions due to their high efficiency in processing high-dimensional data. It works on the principle of finding a best hyperplane to separate interacting protein pairs from non-interacting protein pairs. The best results could obtained when the number of features (such as amino acid composition or sequence motifs) is extremely high relative to the amount of training examples. Lin, Song, Tao, Wang, Wan, Huang, Xu, Chebii, Kitony and Que [49] developed Rice_Phospho 1.0, an SVM-based predictor that achieved 82% accuracy and a Matthews correlation coefficient (MCC) of 0.64. The model uses amino acid occurrence frequency (AF) and AF with composition of k-spaced amino acid pairs (AF-CKSAAP) features for PPI prediction. Karan, Mahapatra and Sahu [55] applied a SVM model to predict PPIs between rice and *Magnaporthe oryzae*, showing high accuracy within this specific system. Murmu, Chaurasia, Rao, Rai, Jaiswal, Bharadwaj, Yadav and Archak [31] introduced PlantPathoPPI, where SVM models trained on Auto Covariance (SVM_AC) and Conjoint Triad (SVM_CT) features achieved >96% accuracies. Such studies illustrate the biological translation of ML predictions: PID2 and OsRac1 were later validated as key components of pathogen-response signaling [79]. Despite high performance, the model’s reliance on small, curated datasets limits its scalability to the full rice interactome.

**Figure 1:**
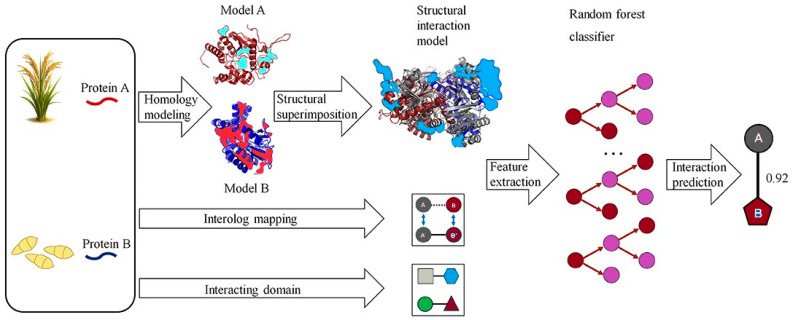
A computational method predicts pathogen–host protein–protein interactions (PPIs) by analyzing protein pairs (A, B). Homology modeling constructs 3D structures, and structural alignment identifies the closest PDB complex template. Four structure-based scores are calculated by overlaying homology structures onto the template. Random forest classifiers integrate structural evidence, homologous mapping, and interacting regions to predict interactions. Modified from Zheng, Liu, Sun, Zhao and Zhang [27], licensed under CC BY 4.0 (https://creativecommons.org/licenses/by/4.0/, accessed on 8 October 2025).

Another traditional ML approach is RF, an ensemble learning method that constructs multiple decision trees during training and outputs the mode of the classes (classification) or mean prediction (regression) of the individual trees. RF has been widely adopted in bioinformatics for its ability to handle large feature sets, resistance to overfitting, and robustness to noisy data. Liu et al. [80] employed a RF model to predict PPIs in rice by integrating features such as domain-domain associations and gene co-expression, achieving an AUC above 0.85. This highlighted RF’s strength in handling heterogeneous biological data. Additionally, RF’s feature importance ranking could facilitate the identification of key species-specific features. Wei et al. [81] introduced a Cascade Random Forests (CRF) algorithm for predicting PPI sites using only protein sequence data. To address class imbalance, their CRF model linked multiple RFs trained on balanced subsets in a layered structure. Each residue was encoded using a 186-dimensional feature vector combining PSSM, averaged cumulative hydropathy (ACH), and predicted relative solvent accessibility (PRSA). CRF-PPI outperformed tools like PSIVER and LORIS across datasets such as Dset186 and PDBtestset164. It also showed robustness to parameter changes and identified PRSA as the most informative feature. While RF and SVM excel in interpretability and handling rice-specific features like PSSM and CKSAAP, they are limited by their dependence on curated datasets and may struggle with capturing complex, nonlinear interactions compared to deep learning (DL) models. For instance, SVM models often require extensive feature engineering, which can be challenging given the sparse experimental PPI data in rice [47]. Figure 1 illustrates a computational pipeline that predicts host–pathogen PPIs by combining homology modeling and structural alignment. Each protein pair (A, B) is aligned to the closest PDB template complex, and four structural scores interface compatibility, electrostatic complementarity, binding energy, and template similarity are computed. These scores serve as inputs to an RF classifier, which integrates homology-based evidence to predict interactions [27]. This approach captures the principle of interolog mapping, where interactions conserved across species inform rice PPI inference. Biologically, it explains how orthologous receptors (e.g., Xa21) and pathogen effectors (e.g., AvrPik) are predicted and later validated as resistance determinants in rice.

k-NN is a powerful but simple non-parametric algorithm used in regression and classification. It predicts the class of a sample by the most common class among its k nearest training samples in feature space. It has been used in biological interaction prediction tasks because of its simplicity and capacity to learn local patterns in data. Although k-NN is seldom used for PPI predictions in rice, it has the potential. Guo et al. [82] applied k-NN for rice protein function annotation based on sequence and expression similarities, suggesting its applicability to PPI via proximity-based feature comparisons. Villikudathil, Jayachandran and Radhakrishnan [74] used a k-NN model with proteomic features to predict rice blast resistance, achieving 90.55% accuracy. The selected markers were linked to plant defense and signaling pathways. However, k-NN has notable limitations, such as sensitivity to irrelevant features and high computational cost for large datasets. Dimensionality reduction techniques or feature selection methods are often required to improve its scalability and accuracy. Table 4 summarizes key traditional ML approaches for rice PPI predictions.

### 3.2. Deep Learning Approaches

Deep learning (DL), such as Deep Neural Networks (DNNs), Graph Neural Networks (GNNs) and Convolutional Neural Networks (CNNs), have emerged as powerful tools for PPI prediction, leveraging their ability to automatically extract hierarchical features from raw data such as amino acid sequences, structural embeddings, and expression profiles. Compared to classical ML models like SVM or RF, DL methods can capture complex, nonlinear relationships and context dependencies, making them especially suitable for modeling the intricate nature of biological interactions. These models are particularly valuable when traditional sequence alignment or structural homology approaches fall short due to the lack of annotated data in non-model crops like rice. Direct applications of DL for rice PPI predictions are still emerging, constrained by limited experimental PPI data compared to other model organisms [89]. However, studies in related plant systems provides a strong basis for rice-specific adaptations.

**Figure 2:**
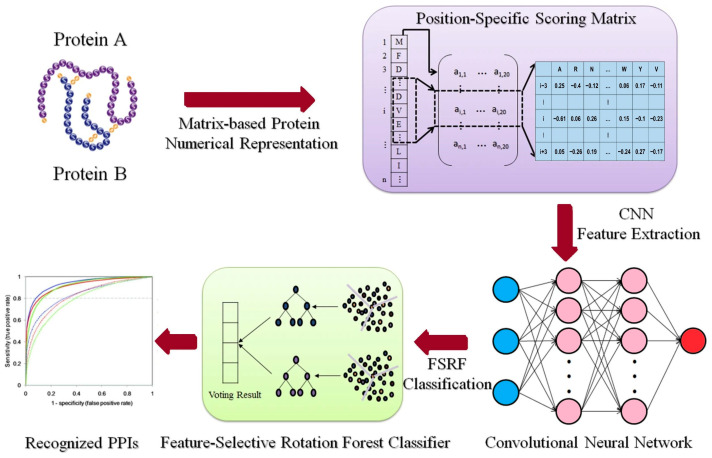
This illustration shows how to combine a feature-selective rotation forest model with a CNN to predict protein–protein interactions, modified based on Wang, Wang, Liu, Yan and Song [93], licensed under CC BY 4.0 (https://creativecommons.org/licenses/by/4.0/, accessed on 8 October 2025).

For CNN-based model, Du et al. [90] developed DeepPPI for general PPI predictions that outperformed traditional methods on multiple datasets. A similar approach could leverage rice-specific datasets like RicePPINet to identify interaction motifs critical for stress response pathways, such as drought or pathogen resistance [47]. Subsequently, Wang et al. [91] introduced another model to predict PPI in *Arabidopsis thaliana*, integrating domain knowledge with raw sequence input. The model was trained on experimentally verified PPIs and achieved an F1-score above 0.9, outperforming SVMs and RFs. Chi, Ma, Wan, Deng, Wu, Cen, Zhou, Zhao, Wang and Ji [33] developed a multi-view GNN model integrating multiple biological networks (expression, functional annotations, and phylogeny) to predict plant PPIs. Their method achieved strong results across several plant species and lays the groundwork for applying multi-model GNNs in rice interactome mapping. Application of these models to rice with datasets like PRIN requires careful model tuning to avoid overfitting [92]. Figure 2 illustrates the process of integrating a feature-selective rotation forest model with a CNN for PPI prediction. In this framework, rotation forest performs dimensionality reduction and feature decorrelation on PSSM descriptors, producing orthogonal feature subsets that capture diverse sequence patterns [93]. The CNN component then extracts spatial and contextual features from these subsets, allowing the model to learn motif-level dependencies associated with interaction interfaces. This integration improves model generalizability and biological interpretability for example, identifying co-evolving residues that mediate protein binding in stress-related networks.

Pan et al. [94] combined DNNs with Discrete Hilbert Transform (DHT)-based signal encoding to predict PPIs across plant species, including rice. In this method, protein sequences were transformed into digital signals via physicochemical descriptors, which were then processed using the DHT to capture frequency-domain features. These enriched signals served as input to a multi-layer DNN. The model achieved high predictive accuracy, with an area under the curve (AUC) of 0.9440 for rice. Further advancing the field, Pan et al. [95] developed another significant DL framework DWPPI for PPI prediction, which integrates network embedding techniques and transforms nodes (proteins) in a PPI network into numerical vectors with DNN to learn nonlinear interaction patterns. The embeddings were derived from various sources, including protein sequences, functional annotations, and expression data. When tested on rice datasets, DWPPI achieved an AUC of 0.9213, indicating its robust performance in inferring novel interactions. This model highlights the value of multi-source data integration.

Zhang et al. [96] applied a GNN model to predict gene–gene interactions using a rice co-expression network, achieving high performance and interpretability. The same principle can be extended to PPI by constructing graph embeddings of protein functional associations and training a GNN to infer unknown interactions. GNNs are particularly promising for rice due to their ability to model network topologies, but their performance depends on high-quality co-expression data, which is often limited for condition-specific rice PPIs [40]. Moreover, Kumar, Khatri and Acharya [53] introduced a DL-based model called DLNet to explore the rice interactome’s dynamic response to pathogen invasion. DLNet was trained on integrated transcriptomic and PPI datasets to model the immune network’s topology under different pathogen challenges. The model revealed pathogen-specific network architectures in rice’s immune response to *Magnaporthe oryzae* (causal agent of rice blast) versus *Xanthomonas oryzae* (bacterial blight). DLNet outperformed conventional ML methods such as RF and SVMs in terms of accuracy and robustness during cross-validation. However, DLNet’s high computational cost and dependence on integrated omics data pose challenges for widespread adoption in rice [97]. Table 5 summarizes the key DL approaches for rice PPI predictions and their related information.

### 3.3. Applications and Case Studies

ML has become a powerful tool in rice functional genomics, providing novel insights into the complex PPI networks that govern diverse biological processes. ML-based PPI predictions enable researchers to model interactomes, which could be applied to identify agronomic trait-associated genes, plant–pathogen interactions, and functional annotation of unannotated genes.

#### 3.3.1. Identification of Candidate Genes for Agronomic Traits

One of the most significant applications of PPI predictions using ML is candidate gene identification for complex agronomic traits such as drought tolerance, salt tolerance, and disease resistance. These traits are governed by complex polygenic networks, and PPI predictions may provide insights into the functions of individual proteins in these networks. For example, Liu, Liu, Zhao, Cai, Qian, Zuo, Zhao and Zhang [80] integrated gene expression data with the RicePPINet interactome to identify drought-responsive sub-networks in rice. Their ML model prioritized proteins involved in ABA signaling, stress perception, and transcriptional regulation. Several of the predicted genes were experimentally validated and are currently under consideration in marker-assisted selection programs aimed at improving drought resilience in rice. Additionally, De Silva, Weeraman, Piyatissa and Fernando [37] applied an ensemble of network algorithms to a seed development PPI network, and predicted 196 new proteins linked to rice seed development. Their analysis revealed 14 functional modules and identified several hub proteins (e.g., SDH1) central to endosperm and seed growth pathways. These hub proteins and modules suggest candidates for improving grain size and yield. Such computational predictions of trait-associated genes, complemented by validation (e.g., transcriptomics or mutant analysis), illustrate how PPI networks can flag candidate genes for breeding.

#### 3.3.2. Understanding Plant–Pathogen Interactions

Plant immunity is largely mediated through protein interactions that detect and respond to pathogen invasion. ML algorithms can predict potential host–pathogen PPIs, providing valuable information about immune signaling pathways and resistance mechanisms. Zheng, Liu, Sun, Zhao and Zhang [27] used a structure-based ML approach to model the rice–*Magnaporthe oryzae* interactome. Their random forest classifier, trained on structural docking features, predicted a novel interaction between the rice immune receptor PID2 and the fungal effector AVR-Pik. This computational prediction was subsequently validated in planta using co-immunoprecipitation assays. This discovery provided a mechanistic explanation for the specific resistance conferred by the PID2 allele and identified AVR-Pik as a direct virulence target, offering a precise genetic module for breeding blast-resistant rice varieties. This case exemplifies how ML predictions can move from in silico discovery to elucidating the molecular basis of pathogen resistance.

Moreover, Karan, Mahapatra, Sahu, Pandey and Chakravarty [83] constructed a comprehensive map of the molecular battlefield between rice and the blast fungus (*Magnaporthe oryzae*) by integrating four distinct biological principles, i.e., interolog mapping, domain interaction, Gene Ontology (GO) semantic similarity, and phylogenetic profiling, into an SVM model. Their approach predicted a massive interactome of 59,430 potential interactions, connecting 1801 rice proteins with 135 fungal effectors and virulence factors. The high accuracy (~95%) of their classifier on known interactions lends credibility to these predictions, which provide a systems-level view of the infection process. The true biological value of this map lies in its ability to pinpoint specific, high-priority targets for functional validation. These predictions suggest a precise mechanism by which the fungus suppresses the plant’s primary immune response. Furthermore, by identifying rice proteins that are targeted by multiple fungal effectors, the model can reveal critical “hubs” in the defense network whose disruption would be most detrimental to the plant. This resource moves beyond a simple list of interactions to generate testable hypotheses about the molecular strategies of infection and defense, ultimately guiding the identification of durable resistance genes and the development of new strategies to counter pathogen effector functions.

#### 3.3.3. Elucidating Salt Tolerance Mechanisms

Salinity is a major abiotic stress that impairs rice growth and productivity. ML-derived PPI networks provide a platform to understand how rice perceives and responds to salt stress at the protein level. Chen et al. [100] developed a novel ML framework, KANMB (Kolmogorov-Arnold Network for Metabolic Biomarkers), to integrate transcriptomic and metabolomic data from the halophyte *Spartina alterniflora*. The model identified 226 salt-responsive metabolites and associated differentially expressed genes, particularly highlighting the flavonoid biosynthesis pathway as a key component of salt tolerance. Functional validation in rice showed that overexpression of a key regulator, SaMYB35, enhanced flavonoid accumulation and salt tolerance under high NaCl conditions. This study demonstrates the translational power of ML in uncovering stress-responsive biomarkers and gene targets, offering a valuable genetic toolkit for improving salt tolerance in rice and other cereals. Furthermore, Pradhan et al. [101] combined an ML-based PPI approach with gene expression profiles from salt-tissues to identify the salt-stricken regulatory network. Their ML predictions illuminated a regulatory network where transcription factors like DREB and NAC central hubs known for activating stress-responsive genes directly or indirectly interact with and regulate proteins involved in Na+ sequestration (e.g., NHX-type antiporters) and K+ retention. This provides a mechanistic model: under salt stress, the predicted PPI network facilitates a coordinated response where stress signals are transduced through DREB/NAC factors to directly modulate the activity of ion transporters, preventing toxic Na+ accumulation and maintaining essential K+ levels within the cell. The results have reported many ongoing breeding programs targeting saline-affected areas. By integrating predicted protein interactions with real-time expression data, the study provided a system-level approach to how rice adapted to salt stress. Major molecular players were not only recognized but also functionally preferred, which provided actionable goals for genetic improvement.

#### 3.3.4. Network-Based Functional Annotation of Uncharacterized Proteins

Despite the availability of a complete rice genome, a large portion of the proteome remains uncharacterized. ML-based PPI networks can facilitate function prediction by associating unknown proteins with annotated ones via shared interaction patterns, semantic similarities, and network topologies. Li, Shi, Zhang, Zeng and Li [48] employed a rule-based DL classifier to predict the function of previously unannotated proteins in the rice interactome. Their model successfully identifies an uncharacterized protein that is a hub node in a sub-network densely populated with annotated kinases and transcription factors known to be involved in the jasmonic acid (JA) signaling pathway. By propagating GO terms across this network, the model would confidently annotate this unknown protein with terms like “JA-mediated signaling pathway” and “defense response to fungus.” This approach successfully annotated numerous rice proteins with putative roles in growth regulation, metabolic control, and stress signaling. These computationally derived hypotheses are invaluable for prioritizing targets for wet-lab validation, such as generating CRISPR knockouts to confirm a predicted role in disease resistance, thereby dramatically accelerating the functional annotation of the rice genome.

#### 3.3.5. Precision Breeding and Genome Editing Target Prioritization

With the increasing use of genome editing tools such as CRISPR/Cas9 for rice improvement, there is a growing need to prioritize functionally relevant targets. ML-driven PPI predictions can identify hub proteins or bottlenecks in signaling pathways, serving as candidates for precise editing. Liu et al. [102] generated a global rice interactome (RiPPID) by a high-throughput Y2H screening pipeline (BIP-seq), mapping >23,000 PPIs (≈22,700 novel) within weeks. Within this network, they identified a “yield module” enriched for yield-related regulators. A transcription factor, bZIP58, appeared as a hub interacting with known yield genes. CRISPR/Cas9 knockout of bZIP58 in rice led to significant reductions in seed number, tiller count, and grain yield, experimentally validating its role. This case shows how network hubs can be rationally chosen as editing targets to improve agronomic traits.

Additionally, Smet et al. [103] applied an ML framework to predict transcriptional responses to drought stress from rice genomic features. Using RF models trained on regulatory elements including promoter motifs (pCREs), transcription factor binding sites (TFBSs), and nucleotide content. The study identified key regulatory elements linked to drought-responsive gene expression. Importantly, the authors employed SHAP interpretability techniques to highlight motifs and sequence features most predictive of gene activation under drought conditions. This approach not only enhances understanding of the regulatory code underlying abiotic stress responses but also provides a rational basis for selecting candidate genes for genome editing to improve drought tolerance in rice.

## 4. Conclusions and Perspectives

It is well known that most proteins do not function in isolation but rather within the context of other proteins. Therefore, the prediction and verification of PPIs are important for a comprehensive understanding of protein functions. ML has revolutionized the prediction of PPI, and it is now feasible to predict and study PPIs at large scale. In rice, all these advances have resulted in unparalleled success in molecular network-based understanding of agronomic traits such as disease resistance, and stress tolerance. Protein interaction prediction and the integration of ML-based predicted protein interactions into gene networks can accelerate the identification of key genes and improve breeding programs aimed at increasing crop performance under biotic and abiotic stress conditions.

However, this progress faces several persistent challenges. The access and quality of training data continue to be major constraints when predicting rice PPIs. With only a few documented rice PPIs in publicly available datasets compared to the available PPIs for Arabidopsis, researchers have the choice of using high-throughput datasets that are often noisy or using interolog-based inference, which contains biases. Compounding this data scarcity, functional annotations are often sparse, limiting the number of features available for model training. Models are often constrained by small dataset size or poor cross-validation procedures that lead to overfitting and inflated performance metrics with no generalizability. Most models also assume PPIs are static structure, and do not account for network rewiring of the interactome and PPIs due to developmental cues. Finally, the “black box” nature of complex models, particularly in deep learning, hinders biological interpretation and validation. These issues are exacerbated by substantial computational barriers, as the resource demands for training sophisticated models like Graph Neural Networks (GNNs) or processing AlphaFold2 structures are often prohibitive for typical research groups.

To overcome these barriers, the strategic use of pre-trained transformer-based protein language models via transfer learning offers a practical path. This approach allows researchers to fine-tune powerful, general-purpose models on smaller rice-specific datasets, drastically reducing computational costs. Furthermore, applying explainable AI (XAI) techniques to these fine-tuned models can illuminate the decision-making process behind predictions, transforming “black box” outputs into interpretable, testable biological hypotheses. Cloud computing and open-source frameworks such as TensorFlow (version 2.20.0) or PyTorch (version 2.7.0) will further democratize access to these advanced methodologies for developing reproducible models.

Building on this foundation, future research should focus on multi-omics data integration to capture the contextual information lost in sequence-based approaches. A particularly promising direction is the incorporation of proteoform-level data into ML models. Proteoforms, arising from PTMs, alternative splicing, or proteolytic processing, introduce critical variability that alters PPI specificity and affinity, particularly in stress-responsive pathways. For example, proteolytic proteoforms are key regulators in plant hormonal signaling, and in rice, stress-induced proteoforms have been observed in cold tolerance mechanisms. Integrating this data through advanced ML frameworks will allow for more accurate modeling of dynamic PPIs, bridging a critical gap in current static predictions.

In parallel, the development of more sophisticated DL models such as transformer-based PLMs, GNNs, and joint sequence–structure models holds great potential for improving generalizability. To ensure these advanced models are evaluated fairly and consistently, we propose the creation of a rice-specific gold-standard PPI benchmark. This dataset, developed through collaboration with databases like RicePPINet and BioGRID, should integrate high-confidence experimental PPIs, curated negative samples, and condition-specific interactions. Standardized evaluation protocols, emphasizing robust metrics like MCC and PR-AUC, will be crucial for meaningful comparability across studies.

Therefore, the combination of these strategies has the potential to transform PPI predictions in rice, enhancing the accuracy, interpretability, and biological relevance of ML models. By bridging computational predictions with experimental validation, such as through co-expression analysis or CRISPR-based gene editing, ML-driven PPI analysis will continue to underpin rice functional genomics and sustainable crop improvement in the face of climate challenges.

## Figures and Tables

**Table 1:** Summary of Key Data Sources for PPI Studies in Rice.

Data Source	Description	Data Coverage	Key Insights	References
STRING	A database of known and predicted protein–protein interaction, primarily derived from experimental data, computational methods, and text mining.	Limited coverage for rice compared to model organisms	Provides a solid ground truth for known PPIs in various species. Offers a global perspective on protein interactions.	[45]
BioGRID	A comprehensive database of biologically relevant PPIs for multiple species, including rice.	Limited for rice but includes experimentally validated data	Contains experimentally validated PPIs and is useful for high-quality, ground-truth data.	[46]
RicePPINet	A rice-specific PPI database compiled by manually curating data from published studies.	Over 8000 rice-specific interactions	Focused on rice, offers insights into the rice-specific interactome and its biological relevance.	[47]
Arabidopsis Homology	Inferred interactions from Arabidopsis that are conserved in rice based on evolutionary relationships between species.	40% of Arabidopsis PPIs detected in rice	Helps expand the rice PPI dataset through homology, especially in conserved pathways like ABA signaling.	[38]
AlphaFold Predictions	AlphaFold’s protein structure predictions for nearly the entire rice proteome.	Nearly complete rice proteome	Predicts potential binding interfaces and protein structures that assist in identifying PPIs. Useful for uncovering interactions in drought-responsive complexes.	[39]
RiceFREND	A co-expression network resource for rice, integrating transcriptomic data to identify potential functional linkages.	Focused on gene expression relationships	Provides functional context by linking co-expressed genes that may interact with each other.	[40]
Proteomic Datasets (MS)	Mass spectrometry-derived proteomic data that reveal direct evidence of protein interactions.	Varies, includes condition-specific interactions	Useful for identifying condition-specific PPIs, such as those during pathogen infection or stress responses.	[41]

**Table 4:** Summary of Machine Learning Approaches for Rice PPI Predictions.

ML Model	Database	No of PPIs (pos/neg)	Features	Performance	Limitations	Unique Aspects	References
k-NN	NCBI, UniProt	~8000	AAC and dipeptide	ACC: 90%, AUC: 0.9	Simplicity of KNN; does not capture complex patterns	predict rice blast disease-resistant genes versus susceptible genes using their encoding protein sequences	[74]
SVM	Custom rice–M. grisea PPI set (interolog/domain inferred)	59,430 (pos only; negatives sampled)	AAC, CT	ACC ≈ 89% (CT features: 89%; AAC features: 88%)	Large predicted set but few true positives for training; limited experimental validation	Integrates interolog-, domain-, GO-, and phylogeny-based models to generate dataset; applies ML on host–pathogen PPIs	[83]
Rotation Forest	PRIN (predicted Rice Interactome Network)	9600 (4800/4800)	PSSM + Discrete Hilbert Transform (DHT)	ACC 94.24%, MCC 0.8914	Random negative pairs may include true PPIs; ignores structure/GOs	First to apply Discrete Hilbert Transform on PSSM for rice PPI prediction; achieves high ACC with only sequence data	[84]
Rotation Forest (ensemble of RFs)	Plant PPI sets (Arabidopsis, maize, rice; from DIPOS/PRIN)	9600 (4800/4800)	PSSM + local optimal-oriented pattern (LOOP)	ACC 94.02% (RF: 90.90%, SVM: 88.95%)	Balanced dataset but possible false negatives; not cross-species tested	Novel use of LOOP descriptor on PSSM with Rotation Forest; high AUC (≈0.96)	[56]
Rotation Forest	PRIN, agriGO	~9600 (various)	PSSM + Discrete Sine Transform (DST)	ACC 88.82% (rice)	Lower accuracy on rice vs. other plants; depends on dimensionality reduction (SVD)	Introduced DST on PSSM for plant PPIs; shows efficacy of signal-processing features	[85]
Rotation Forest	PRIN	~9600 (various)	PSSM + Fast Walsh–Hadamard Transform (FWHT)	ACC 94.42% (rice)	Computationally intensive FWHT; relies on high-quality PSSMs	Applies FWHT to extract features from PSSM for ensemble classification; very high accuracy on rice data	[86]
RF	HPID	2018 (structure-matched pairs)	Structural docking scores, compatibility	N/A (focus on network discovery)	Relies on availability of structural templates; no standard ML metrics reported	First 3D-structure-based PPI predictor for rice–pathogen; built an RF classifier on docking features	[27]
SVM	Predicted Rice–M. grisea PPIs (interolog/domain)	532 (pos only; negatives sampled)	AAC, CT (sequence composition)	Jackknife ACC 93.85%	Very small dataset (532); no independent test set beyond 22 pairs; potential overfitting	Combined interolog and domain inference to generate positive PPIs, then SVM to classify; enriched predicted network with pathogen effectors	[87]
Gray BPNN	Not specified	1356	AAC (sequence feature)	ACC = 92.78%	Difficulties to handle large-scale dataset	Demonstrated feasibility of neural network for rice PPI with low computational cost	[88]

**Table 5:** Summary of Deep Learning Approaches for Rice PPI Predictions.

DL Model(s)	Database	No. of PPIs	Features	Performance	Limitation	Unique Aspects	References
Ensemble of Siamese RCNN (sequence), Domain2vec MLP, GO2vec MLP + logistic regression	Arabidopsis PPI dataset (BioGRID) with curated negatives	Not specified	Sequence embeddings (word2vec → RCNN); domain embedding (domain2vec); GO term embedding (GO2vec)	Cross-species (Arabidopsis → rice) AUC not reported here, but claimed “better than ML methods, though overall remains to improve”	Requires high-quality GO/domain annotations; cross-species performance still limited	Multi-view ensemble (sequence + domain + GO); Siamese RCNN captures pairwise sequence interaction; provides web server for Arabidopsis → rice PPI prediction	[44]
Pre-trained Transformer (ESM-1b) + MLP (ESMAraPPI)	Arabidopsis PPI (BioGRID) with strict train/test splits	Not specified	Protein language model embeddings (ESM-1b) for each protein sequence	AUPR ~0.810 on strict independent set (no rice test reported)	Focused on Arabidopsis only; requires large pretrained model; no cross-species evaluation	First use of large pre-trained protein transformer (ESM-1b) for plant PPI; shows strong extrapolation (unseen proteins); outperforms other pLMs and baselines	[98]
DeepWalk graph embedding + 4-mer word2vec + DNN classifier (DWPPI)	PRIN (rice) and PPIM (maize) databases	Rice: 103,028 (positives)	Sequence (word2vec on 4-mer tokens) + network-behavior (DeepWalk embedding of PPI graph)	Rice AUC ≈ 0.9213	Requires existing large PPI network for embedding; performance may drop on sparse networks	Multi-source fusion (sequence + network) for plant PPIs; large-scale (100K + PPIs) datasets; case studies validated top predictions against literature	[95]
DCSE: Siamese CNN + BiGRU ensemble (double-channel)	Human PPI (STRING/HPRD)	~30,000	NLP-based sequence encoding (skip-gram) + CNN, BiGRU	Acc 93.0%, Precision 90.9%, Recall 94.5%, F1 92.7%, MCC 0.860	Human-specific training; no plant evaluation; uses large one-hot embeddings	Novel siamese-ensemble architecture (parallel CNN and CNN + BiGRU); robust to imbalanced data	[99]
DLNet	STRING, PRIN, IntAct	~20,000	Sequence similarity	Precision: 90%, Recall: 84%	High false positives	Uses both the forest model and graph-embedded deep-forward network (GEDFN)	[53]
DNN combined with Discrete Hilbert Transform (DHT)	PRIN Rice PPI set (4800 pos, 4800 neg)	4800	PSSM (via PSI-BLAST) + DHT of PSSM (followed by SVD)	Rice AUC ≈ 0.9440 (Acc 82.6%, F1 ≈ 85%, MCC 0.676)	Relies on sequence alignments (PSI-BLAST) for PSSMs; uses negative sampling (non-validated negatives); only sequence features	Innovative use of DHT on PSSM to capture evolutionary info; strong cross-plant evaluation	[94]
DeepPPI: Fully connected DNN	Yeast PPI (DIP) positives vs. sampled negatives	~6600	PSSM (evolutionary profile) + other sequence descriptors	Accuracy ≈ 65.8% (vs. 64.2% by SVM)	Moderate accuracy; only yeast data; no plant test	One of the first DNN models for PPI; showed slight improvement over SVM	[90]

## Data Availability

No data was used for this research.
